# The obstacles to organ donation following brain death in Iran: a qualitative study

**DOI:** 10.1186/s12910-020-00529-8

**Published:** 2020-09-01

**Authors:** Parvin Abbasi, Javad Yoosefi Lebni, Paricher Nouri, Arash Ziapour, Amir Jalali

**Affiliations:** 1 Department of Nursing, School of Nursing and Midwifery, Kermanshah University of Medical Sciences, Kermanshah, Iran; 2 Research Institute for Health, Kermanshah University of Medical Sciences, Kermanshah, Iran; 3 Department of Midwifery, School of Nursing and Midwifery, Kermanshah University of Medical Sciences, Kermanshah, Iran; 4 Ph.D. Student, Health Education and Health Promotion, Health Institute, Kermanshah University of Medical Sciences, Kermanshah, Iran; 5 Substance Abuse Prevention Research Center, Research Institute for Health, Kermanshah University of Medical Sciences, Kermanshah, Iran

**Keywords:** Brain-death, Organ donation, Obstacles, Qualitative study

## Abstract

**Background:**

Organ donation following brain death has become an important way of supplying organs for transplantation in many countries. This practice is less common in Iran for different reasons. Therefore, this study aims to explore the obstacles to organ donation following brain death in Iran.

**Methods:**

This qualitative research was conducted following the conventional content analysis method. The study population consisted of individuals with a history of brain death among their blood relatives who refused to donate the organs. Snowball sampling was employed to select the participants. In-depth semi-structured interviews were conducted for data gathering. Theoretical saturation was achieved through 20 interviews. Data analysis was done following the steps proposed by Graneheim and Lundman. Lincoln and Guba’s criteria were used to ensure data rigor and transferability of the study.

**Results:**

Data analyses revealed 185 codes, 23 categories, and seven themes including, poor knowledge about brain death and organ transplantation from a dead body, cultural beliefs, religious beliefs, deficiencies of requesting process, fear and concerns, inability to make a decision, and social learning.

**Conclusion:**

There were several factors in families’ reluctance to donate organs of a brain-dead patient. Through improving knowledge and changing cultural beliefs in society, it is possible to take large steps towards promoting organ donation from brain-dead patients.

## Background

Brain death happens when all the brain functions are stopped and an irreversible brain damage takes place [1]. Organ donation is an altruistic decision that can be made by the family members after brain death [2]. Although, many organizations and medical centers have implemented various interventions and training courses to increase satisfaction with organ donation [3, 4], a lack of organs for donation still is a serious problem in the world [5]. As a result, thousands of patients on waiting lists for transplantation die every year [6].

Iran is one of the countries with a shortage of donated organs [7]. On average, there are 2500–4000 brain deaths per year in Iran which can be candidates for organ donation. However, only 926 families of brain-dead patients consented to organ donation in 2017. The organ donation rate in Iran is 10.9 per one million and this rate places Iran at the 27th rank in the world [8].

Reluctance to donate organs is affected by several factors like attitudes toward organ donation [9], religious beliefs [10–13], incorrect perceptions of brain death [14, 15], dissatisfaction with the care system [16], lack of trained staff to negotiate with the family of the brain-dead patient [17], the family’s desire to keep the patient’s body intact [18], and education level [19]. Studies have shown that the perception of brain death and the decision to donate organs is related to the individual who makes the request, the timing of the request, and the way of expressing the request [20]. A study by De Groot et al. showed that improper timing to make the request for organ donations, lack of support by relatives and medical team, and inadequate knowledge about organ donation were the factors contributing to refusals to organ donation by family members [21].

Sotillo et al. (2009) highlighted disagreements among family members regarding organ donation. They reported that family members were very much concerned about the delivery of the donated organ to the individuals in need, concerned about deformation of the dead body, and reluctant to accept brain death, which were the main obstacles to organ donation [22]. Siminoff et al. (2007) showed that altruism, learning about the patient’s desire to donate their organs after death, giving emotional support to families with a patient who needs an organ transplant, and provision of adequate information about the organ donation process were the factors in the family’s decision to donate organs [23]. Rodrigue et al. (2008) highlighted disagreements among family members based on the characteristics of the brain-dead patient, attitudes toward organ donation, and dissatisfaction with the medical team’s services [24].

Since different societies, depending on their values, beliefs, and cultures, have different ways of dealing with brain death and securing consent organ donation, it is essential to examine this issue in Iranian society. The majority of studies in this field have been conducted following experimental and qualitative approaches [25–28]. Moreover, few studies have been conducted on the obstacles to organ donation following brain death in Iran. Therefore, a qualitative examination of this complicated and multi-dimensional phenomenon is necessary. This qualitative study is an attempt to elaborate on the obstacles to organ donation following brain death in Iran.

## Methods

### Design of the study and selection of participants

This study was carried out using a qualitative approach and conventional content analysis following Graneheim and Lundman’s approach [29]. Inclusion criteria included having a history of brain-dead patient capable of donating organs in blood relatives, refusal to donate an organ, and desire to express experiences and participate in the study.

### Data collection

The interviews were started by guide questions like, What happened after the brain death of your family member?; What did you do then?; What were your perceptions and the mental image of brain death and organ donation?; Why did you not give consent to donate an organ?; What was the relatives’ role in your decision?, How did you find the medical personnel’s behavior in asking for organ donation?; and so on. The interview guide questions were developed specifically for this study. The interviews were followed by probing questions to identify the obstacles to organ donation. The time and place of interviews were chosen by the participants, occurring mostly at their houses or other places like workplaces or public parks. The data collection process took place from August 2019 to December 2019, and interview time ranged from 50 to 90 min. The interviews were recorded by the voice recorder with the permission of the participants. The researchers conducted interviews individually and selected one person from each family. The participants were influential and decision-making members of the family, though not necessarily the primary decision-maker.

The purposeful and snowball sampling method was used to select the participants and continued until theoretical saturation was achieved. Theoretical saturation was achieved after conducting 20 interviews. No new code was extracted after the 16th interview; however, to avoid false theoretical saturation, four further interviews were conducted in which no new code was extracted.

### Data analysis

The data was processed following the five steps proposed by Graneheim and Lundman [29], so that all the interviews were transcribed at first by two researchers, and then the whole texts were reviewed frequently to achieve a general picture of the interview outcomes. Then, the researchers categorized the interviews into semantic units and primary codes. Afterwards, the similar primary codes were grouped into general categories, and as the final stage, categories and themes were extracted. The researchers tried to have the highest homogeneity and heterogeneity within and among the categories, respectively.

### Rigor

To ensure rigor and transferability, Lincoln and Guba’s techniques were used [30]. To improve credibility, the researchers had a long-term and continuous engagement with the participants and the study setting. The participants received the data analysis results for confirmation. Another method to improve the credibility of the data was to select participants with different traits, which added to richness of the concepts. Conformability was ensured by leaving the prejudices and perceptions aside and respecting the anti-bias approach in data gathering, analyzing, and publishing the findings. Besides, experts who were familiar with and had different viewpoints about the subject were asked for their opinions. Parts of the interviews and the codes and categories were sent to a few physicians and nurses, who were in contact with the families of brain-dead patients for feedback. The interviews were transcribed and encoded by two team members to check the dependability and stability of the findings. The findings were shared with colleagues and non-colleague researchers for feedback. For transferability, a deep and detailed account of the study setting and participants was prepared. Moreover, the demographics of participants were provided, along with plenty of direct quotes.

Consistent with ethical standards, the participants expressed their consent with voice recording. The interviews were held in a peaceful environment. The confidentiality of information was maintained, and the participants were informed that they could leave the study at whatever stage and that they could have access to the results.

## Results

The research team conducted 20 interviews. The demographics of the participants are listed in Table 1. The analyses revealed 185 codes, 23 categories, and seven themes (Table 2). In total, 185 primary codes were truncated by removing codes with similar meanings.

**Table 1:** Demographics

Variable	Dimensions	N
Age group	< 20	4
	21–40	7
	> 40	9
Gender	Male	12
	Female	8
Relationship with the patient	Father or mother	7
	Brother or sister	6
	Wife	3
	Child	4
Educational level	Uneducated	2
	Middle school	8
	High school	10
Causes of brain death	Accidents	10
	Fall from height	6
	Drown in water	2
	Stroke	2

**Table 2:** Themes and Categories

Themes	Categories
Inadequate knowledge about brain death and organ transplantation from a dead body	Lack of knowledge about brain death Lack of knowledge about the benefits of organ donation Lack of knowledge about organ donation process
Cultural beliefs	One’s soul may not rest in peace if the whole corpse is not buried Opening a human’s body is disrespectful to the corpse One is alive as long as the heart is working and the body is warm
Religious beliefs	If one’s organs are transplanted into another person, all the sins of the receiver will be counted as the donor’s sins. Killing a soul is forbidden by the religion (Haram) Fatalism Belief in miracle
Deficiencies of requesting process	Lack of a sympathetic atmosphere Lack of counseling services about organ donation Lack of adequate mental support by the relatives Lack of persistence for organ donation
Fear and concern	Fear of regret Fear of others’ reaction Fear of future tensions in the family
Inability to make decision	Limited time to think Disagreement among the family members Lack of knowledge about the brain-dead patient’s attitude about organ donation Lack of trust in the medical personnel
Social learning	Lack of public information efforts about organ donation by the media Not a common practice No similar case happened to relatives and friends

### Themes of study

#### Inadequate knowledge about brain death and organ transplantation from a dead body

Many of the participants were not familiar with the concept of brain death. Therefore, it was not easy for them to realize what happens to the body after brain death. They also did not know much about the organ donation process. One participant said:

**“***We thought that he might return, like in movies that patients in coma wake up. That is why we did not give our consent to organ donation….” (P3).*

*“To be honest, I had no idea how many lives we could have saved by donating my brother’s organs” (P6).*

*“I thought that organ donation was useless, and by that, we would only create more pain for our patient….” (P17).*

*“We thought that they would cut his body into pieces if we gave our consent for organ donation. If so, how we could wash his body and hold a proper funeral…” (P8).*

*“I thought we should wait for a long time, and every day they would remove one organ of his body. It did not sound like a good idea to me so I decided to reject their request…” (P7).*

Since there is not enough public education about brain death and the necessity and process of organ donation in Iran, many do not have a clear understanding of it. Consequently, many families have incomplete and even wrong information about organ donation, which leads to their reluctance to donate organs.

#### Cultural beliefs

A set of cultural beliefs about death and the corpse creates ambiguities in Iranian families about organ donation. Some of the participants noted in this regard:

“*I have heard, if the whole body is not buried in one place, the soul will suffer badly in the other world. Of course, we did not want this for our patient…” (P3).*

*“I think it is disrespectful to the deceased to open his/her body and take out the organs…” (P8).*

*“His body was still warm; I could not make myself donate his organs…” (P20).*

*“When the heart beats, it means that the person is alive. So, it is not possible to donate the patients’ organs” (P19).*

According to the results, for many, a person is considered dead when the heart stops beating, and the body becomes cold. Therefore, it was not easy for the participants to accept brain death as a sort of death and give their consent for organ donation. In addition, some participants believed that it was disrespectful to the deceased if some parts of the body are not buried.

#### Religious beliefs

All the participants were Muslims, and their beliefs about life and death were an obstacle to organ donation. Based on their religious beliefs, the participants noted:

*“If my husband’s organs were transplanted into another body, all the sins done by the receiver of the organ would be counted as my husband’s sins. That is why I did not give my consent to organ donation…” (P2).*

*“My mother is very religious and we could not convince her to donate my brother’s organ. She said that it is a sin to be the cause of someone’s death…” (P12).*

*“I had hope until the last moment. I think I was waiting for a miracle and that everything would be Ok. But I was wrong; still, I was not able to donate the organs…” (P5).*

The majority of participants believed that human limbs will testify for or against the person in the Last Judgement Day. If one’s limb is donated, all the sins of the receiver of the organ will be counted as the donator’s sins. That was a great obstacle to organ donation. Moreover, according to Islamic trainings, doing any harm to a person who is still alive is forbidden. Many of the participants believed that creating the condition for the death of their patients was prohibited by their religion and considered as a major sin. Additionally, many of the participants believed that death is in God’s hand. Some participants hoped that their patients might come back to life, and some even expected a miracle. Therefore, organ donation from these participants’ viewpoints was an interference in God’s business and a sin as well.

#### The inefficiency of the requesting process

A brain-dead patient creates a complicated situation for the family members as they feel downhearted on one hand and have to decide on organ donation on the other hand. Therefore, the process of requesting for organ donation is very sensitive. An improper way or time for making the request by the medical team might upset the family members and lead to a refusal of organ donation. Some statements in this regard are as follows:

*“The medical personnel treated us badly as if they had no idea what we were going through. The only thing they wanted from us was to donate organs. They were not able to feel our condition…” (P16).*

*“We did not know much about organ donation, and it was unclear for me. There was no counselling service in the hospital...” (P1).*

*“It is very hard to donate your loved one’s organs to another person. You will feel a lot of pressure if you accept it; therefore, we needed a lot of mental support, but there was none…” (P9).*

*“They only asked for organ donation once. We expected that they might ask us again, but they did not. It appeared to us that the hospital was not that much interested in organ donation. Maybe if they had been more persistent, we would have given the consent…” (P15).*

The majority of participants highlighted the lack of counselling services, absence of sympathy, and lack of persistence in the officials as the main reasons for refusing to donate organs. All participants highlighted lack of mental support as well.

#### Fear and concern

Donation of an organ is not a common practice, and the majority of people in the society have negative attitudes towards it. Therefore, most of the participants had concerns about being blamed or judged by others if they had agreed to donate organs. The family members experienced severe fear and doubt, which were a great obstacle to organ donation.

“*I knew that it was over, but I thought that I might regret it. There was nobody to support me…” (P19).*

*“Most of us were OK with the idea, but there was a fear that others might say things behind our backs like ‘they donated organs to get rid of the body…’” (P11).*

*“We were concerned that others might say we had sold his organs. We even heard some of the gossip, and thus, decided to refuse the request for donation…” (P6).*

*“Most of us were ready to accept the request, but we were concerned about future quarrels and altercations at home…” (P13).*

Some of the participants were concerned about feeling regret in the future, and therefore, refused to donate organs. In fact, the participants feared the consequences of their decision to donate their patients’ organs.

#### Inability to make decision

This category refers to the issues that make decision-making on organ donation hard for the families. The majority of brain-death cases in this study were related to car accident victims or falling from a high altitude. Such accidents happen suddenly and traumatize the family members. Thus, it is not easy for them to accept the situation and make a decision on organ donation. Most of the Iranian families are extended families in which relatives like uncles and aunts play a significant role in decision-making. This adds to the complexity of deciding on organ donation. In some cases, the patient did not have an organ donation card and their families were not familiar with organ donation, this made it harder for the family to make decision about organ donation. Some of the participants believed that the donated organs would be sold to the rich. Consequently, it adds to the complexity of decision-making on organ donation. Some of the comments in this regard are as follows:

*“Everything happened in the twinkle of an eye. Our patient was a car accident victim, and it was not easy for us to donate his organs. We were not able to make the right decision, maybe if we had more time, we would have agreed…” (P4).*

*“My father and I agreed with organ donation, but my sister and mother disagreed. Their disagreement stopped us from donating organs…” (P4).*

*“The fact that our patient had not registered as an organ donor, made it hard for us to make the decision. It would have been easier for us if he had an organ donation ID…” (P9)*

*“I have heard that rich and influential people have more chance to receive an organ and the poor have no chance. Because of this, I do not feel good about the idea…” (P11).*

Factors like limited time to make a decision, disagreement among family members, and lack of trust in the medical team made the decision-making process harder.

#### Social learning

Most of the participants stated that they had no role model in their relatives or the media for organ donation. This was a reason for not giving consent to organ donation.

“*Maybe with a bit more organ donation campaigns by the media, we would have made a different decision…” (P8).*

*“We have had a few cases of brain death in our family, and none of them have donated organs, so why should we…” (P15).*

*“Many people suffer from brain death on a daily basis, and most of them do not donate organs, we are just another example…” (P17).*

Despite the high rate of brain death in Iran and the shortage of organs for donation, the media (state-run or private) have failed to cover this issue so that there are few promotional materials in this regard. As a result, many people are unfamiliar with the idea. Furthermore, the majority of participants said that they had no history of organ donation among their relatives and friends. Following the role model of their relatives and friends, they were reluctant to donate organs.

## Discussion

The obstacles to the organ donation following brain death in Iran were ascertained using a qualitative approach. As the results show, the participants had limited knowledge about brain death, and this was a big obstacle. This finding is consistent with previous studies in this field [14, 31]. Since many of the participants did not have a clear definition of brain death and the prognosis of their patients, they did not accept the death of their patients. Consequently, they were not able to give consent to donate organs. Therefore, it is necessary to familiarize the society with the concept of brain death. It is also important to raise public awareness about the process and importance of organ donation. This helps family members of the brain-dead patients to make better decisions regarding organ donation when it is needed.

Lack of knowledge about the benefits and necessity of organ donation was another obstacle to organ donation. Saleem showed that knowledge about brain death, organ donation, and positive attitudes in this regard were significantly related to giving consent to donate organs [32]. The media in Iran barely cover the issue of organ donation, and promotional campaigns are scarce. Therefore, people have a limited knowledge about this issue.

Another main obstacle to organ donation was cultural beliefs. Attitudes and cultural traditions are the key factors that affect people’s behavior about organ donation [9, 33]. Wong reported that showing respect to a brain-dead patient and the body was one of the main issues for Chinese and Indian participants [34]. In Iran, people believe that the soul cannot rest in peace if the whole body is not buried; therefore, many refuse to donate the organs of a dead person. Taking into account the cultural differences of societies, specific measures based on specific cultural beliefs in different societies should be taken to increase the rate of the consent to donate organs without challenging ethical norms in the society.

Religious beliefs were another obstacle to organ donation. Other studies have also shown that religious beliefs in Muslims are an obstacle to organ donation [34, 35]. The set of beliefs about death and the afterlife in Islam convince many Muslims that organ donation is a sin and interference in God’s business. Zhang reported that Confucius’s beliefs about the body were obstacles to organ donation in China [36]. However, according to great Islamic clerics, Islam does not forbid organ donation, and there is no such limitation in Islam [37, 38]. The key restriction is individuals’ knowledge about Islam as they do not know that donating an organ to save other patients’ lives is completely acceptable from a religious point of view. Many Muslims may not be willing to donate the heart or other organs of their dead patients to a needy patient to save a life only because of their lack of knowledge about Islamic clerics/Ulema’s fatwas or verdicts. Therefore, Muslims need to know about Islamic principles and clerics’ opinions or fatwas about organ donation to save lives. In this way, the Muslims who donate organs to needy patients can feel satisfaction about their decision to donate their dead patients’ organs.

Dissatisfaction with the way of making the request was another obstacle to organ donation. Rodrigue et al. and Merchant et al. showed that the way of making the request and the medical team interactions with the family had a notable impact on the decision made by the families [20, 39]. A study by Sque et al. indicated that dissatisfaction with the care system was one of the reasons for rejecting the idea of organ donation [16]. Therefore, providing education to the medical personnel about communicating with family members and creating a proper atmosphere before requesting organ donation from families with a brain-dead patient are recommended to increase the rate of organ donation.

Lack of persistence in the medical personnel was another obstacle to organ donation. The medical personnel can play a significant role in preparing the ground for winning the consent of family members for organ donation. Given the fact that most of the participants believed that the medical personnel were not persistent enough in their request, one may say that probably even the personnel do not support organ donation.

Another obstacle found in the study was the lack of psychological support to the family members. Family members of brain-dead patients experience harsh conditions, and learning about the idea of organ donation adds to their anxiety and stress [40–42]. It is essential to provide adequate mental support for them to help them overcome the stress and make a better decision. A study by Manzari et al. also highlighted the necessity of mental support as an essential need for the family members of brain-dead patients [7].

Fear and concern were other obstacles to organ donation. Many of the participants believed that they would have been blamed by others if they had allowed organ donation. Therefore, making a decision in this regard was not easy for them. Cultural activities to create more positive attitudes towards organ donation can be helpful. Instead of being blamed for organ donation, such families should be supported and celebrated by society.

The inability to make a decision was another category found in this study. Reaching agreement among relatives about organ donation was also mentioned by De Groot et al. as a challenge for organ donation [43]. It is not a simple step to take, and some or all family members might change their minds afterwards, which can lead to tension in the family. Therefore, it is essential to introduce family-centered programs to raise awareness and knowledge about reactions to brain death and organ donation.

Lack of time to think about the request is one of the obstacles to organ donation, which is also mentioned by other studies [43, 44]. This can be due to the special situation that the patient’s family members experience. Disagreement among family members was one of the obstacles to organ donation that was found in this study. This finding is consistent with previous studies in this field [17, 22, 23]. This can be explained by the specific cultural and social beliefs of Iranian society. Many families in Iran have kept their traditional cultural beliefs, and there are strong family ties among family members. This makes it hard to make such decisions. Ahmadian et al. showed in a study that making a decision about organ donation, when there is a disagreement among family members, creates intense stress and pressure [45]. The fact that many brain-dead patients do not have an organ donation card makes it even harder for the family members to make the decision. A donation card facilitates the decision-making process for the family members. Rrunning campaigns for receiving a donation card can solve these problems to a great extent.

The lack of trust in the medical team members was one of the obstacles to organ donation. This finding is consistent with DeJong et al. [46]. The participants did not trust the physician’s opinions and recommendations. In some cases, they were not sure whether the donated organs had been given to the right individuals or not. De Moraes showed that establishing a decent and constructive relationship based on trust between the medical team and the family members of brain-dead patients can improve the chance of giving consent to organ donation [47]. Therefore, it is necessary to clarify the process of organ donation and assure the families that the donation process is a completely fair process that delivers the donated organs to the right individuals.

The next category was social learning, which is a key finding in this study. One may say that families of brain-dead patients have rarely seen or heard of similar experiences in their relatives or on the media about organ donation. That is, the families learn that they should not donate an organ in a similar situation. Therefore, public promotion by the media and highlighting the cases of organ donation, as a public education effort, can change attitudes in the families towards organ donation.

Following a qualitative approach and for the first time, the present study comprehensively examined the obstacles to organ donation from the viewpoint of individuals and families that refused to donate organs of their brain-dead patients. Comprehensive and novel information can be found in this study by national and local decision-makers to introduce an integrated and comprehensive program to overcome the obstacles and facilitate the organ donation process. The diversity of participants was another advantage of the study as the participants were selected from different social and economic backgrounds, which adds to the richness of the findings.

A striking challenge in the present study was finding the subjects, which was solved by receiving support from directors and personnel of hospitals and the cooperative attitudes of the participants to introduce similar cases. The reluctance of the participants to participate in the study was another limitation of the study. This was overcome by introducing the research objectives and assuring them of the confidentiality of their information. The participants were assured that the data would only be used for the research objectives.

## Conclusion

Several factors were found to contribute to the reluctance of family members of brain-dead patients to give consent to organ donation. The most important of them were lack of knowledge, social learning, cultural composition, and religious beliefs. It is essential, therefore, to raise awareness and knowledge in society about brain death and the necessity and condition of organ donation, alter the religious and cultural beliefs about the issue, facilitate the mental and social condition by providing counselling services and cultural activities, and provide mental and social support to the families. In addition, ethical issues such as giving priority to the patients more in need, preventing abusive use of the donated organs such as smuggling or selling them in the black-market, and ensuring the transparency of the organ donation process are recommended.

## Data Availability

The datasets using in the study are available from the corresponding author on reasonable request.

## Abbreviations

BDP: Brain-dead Patients
OOD: Obstacles of Organ Donation
QS: Qualitative study
